# A Germany-wide survey study on the patient journey of patients with hereditary angioedema

**DOI:** 10.1186/s13023-020-01506-5

**Published:** 2020-08-26

**Authors:** Markus Magerl, Holger Gothe, Simon Krupka, Anja Lachmann, Christoph Ohlmeier

**Affiliations:** 1 Dermatological Allergology, Allergie-Centrum-Charité, Department of Dermatology and Allergy, Charité – Universitätsmedizin Berlin, Luisenstraße 2, 10117 Berlin, Germany; 2 IGES Institut GmbH, Friedrichstraße 180, 10117 Berlin, Germany; 3 Shire Deutschland GmbH, Friedrichstraße 149, 10117 Berlin, Germany; 4 Chair of Health Sciences / Public Health, Medical Faculty “Carl Gustav Carus”, Technical University Dresden, Loescherstrasse 18, 01307 Dresden, Germany; 5 Institute of Public Health, Medical Decision Making and Health Technology Assessment, Department of Public Health, Health Services Research and Health Technology Assessment, UMIT – University for Health Sciences, Medical Informatics and Technology, Eduard Wallnoefer Zentrum 1, A-6060 Hall in Tirol, Austria

**Keywords:** Hereditary angioedema, Diagnostic delay, Germany, Rare disease, Survey

## Abstract

**Background:**

Hereditary angioedema (HAE) is a rare genetic disease and characterized by clinical features such as paroxysmal, recurrent angioedema of the skin, the gastrointestinal tract, and the upper airways. Swelling of the skin occurs primarily in the face, extremities and genitals. Gastrointestinal attacks are accompanied by painful abdominal cramps, vomiting and diarrhea. Due to the low prevalence and the fact that HAE patients often present with rather unspecific symptoms such as abdominal cramps, the final diagnosis is often made after a long delay. The aim of this German-wide survey was to characterize the period between occurrence of first symptoms and final diagnosis regarding self-perceived health, symptom burden and false diagnoses for patients with HAE.

**Results:**

Overall, 81 patients with HAE were included and participated in the telephone-based survey. Of those, the majority reported their current health status as “good” (47.5%) or “very good” (13.8%), which was observed to be a clear improvement compared to the year before final diagnosis (“good” (16.3%), “very good” (11.3%)). Edema in the extremities (85.2%) and in the gastrointestinal tract (81.5%) were the most currently reported symptoms and occurred earlier than other reported symptoms (mean age at onset 18.1 and 17.8 years, respectively). Misdiagnoses were observed in 50.6% of participating HAE patients with appendicitis and allergy being the most frequently reported misdiagnoses (40.0 and 30.0% of those with misdiagnosis, respectively). Patients with misdiagnosis often received mistreatment (80.0%) with pharmaceuticals and surgical interventions as the most frequently carried out mistreatments (65.6 and 56.3% of those with mistreatment, respectively). The mean observed diagnostic delay was 18.1 years (median 15.0 years). The diagnostic delay was higher in older patients and index patients.

**Conclusions:**

This study showed that self-perceived status of health for patients is much better once the final correct diagnosis has been made and specific treatment was available. Further challenge in the future will still be to increase awareness for HAE especially in settings which are normally approached by patients at occurrence of first symptoms to assure early referral to specialists and therefore increase the likelihood of receiving an early diagnosis.

## Background

Orphan diseases or rare diseases are defined as life-threatening or chronically debilitating diseases with a prevalence of 1.0 to 7.5 per 10,000 inhabitants (EU 5.0, US 7.5, Japan 4.0, Australia 1.0 per 10,000). For Germany, the number of persons suffering from a rare disease is estimated at four million people [1]. Due to this low prevalence and the fact that the diseases are commonly characterized by a combination of unspecific symptoms, making a clear diagnosis is often challenging. In many orphan diseases the time between first occurrence of symptoms and reliable diagnosis (diagnostic delay) amounts to years. Within this diagnostic delay, symptoms are often attributed to more common diseases which leads to incorrect diagnosis and potentially mistreatment [2, 3]. Studies show that diagnostic delay is associated with anxiety, frustration and stress [4]. In addition to negative effects on individuals´ health and well-being, orphan diseases also have major economic implications including direct costs for medical treatment [5].

Hereditary angioedema (HAE) is a rare genetic disease and characterized by clinical features such as paroxysmal, recurrent angioedema of the skin, the gastrointestinal tract, and the upper airways [6, 7]. HAE is caused by a genetic deficiency or reduced functionality of a protein called C1-Inhibitor (C1-INH). As a result of C1-INH deficiency or limited functionality, excessive bradykinin generation leads to increased permeability of blood vessels, followed by the formation of angioedema [8]. The minimal prevalence of HAE is estimated at 1.5 individuals per 100,000 people [9]. In Germany, approximately 1200 people suffer from HAE [10]. Swelling of the skin occurs primarily in the face, extremities and genitals. Gastrointestinal attacks are also typical and accompanied by painful abdominal cramps, vomiting and diarrhea [7]. Because of the permanent risk of laryngeal swelling and the possibility of suffocation, reduction of the diagnostic delay as well as a timely start of disease-specific therapy is of particular importance in HAE. Most commonly, the first manifestation of symptoms falls in the first or second decade of life [6]. However, delays in diagnosis are considered common in patients with HAE [11]. According to the Icatibant Outcome Survey (IOS) the diagnostic delay for patients with HAE is on average 8.5 years [7]. The IOS registry was a requirement that was issued when Bradykinin B2 receptor antagonist Icatibant was approved by the EMA and was set up as a multinational European registry study. The registry covers over 60 centers in 14 countries with a total of more than 1500 patients enrolled. During the existence of the registry, some centers have closed. Eleven countries are currently participating [12]. Increasingly, research efforts concerning rare diseases such as HAE are being made. However, these research activities are mainly directed at treatment of the disease after final diagnosis. Even though, there are also studies that explicitly deal with diagnostic delay within the framework of large-scale survey studies such as the EurordisCare studies, delay in diagnosis as well as health care situation in HAE has not been examined extensively. In the present work, a survey has been developed to characterize the period between occurrence of first symptoms and final diagnosis for HAE-patients in Germany. This publication is part of an overarching project on rare diseases, the “VISIBL Patient Journey”, which also addresses lysosomal storage diseases (LSD).

## Methods

### Study design and patients

The study was conducted in the design of a telephone based cross-sectional survey. All patients with confirmed diagnosis of HAE were potentially eligible to participate in the study. Diagnosis of HAE was confirmed by self-report. No further inclusion or exclusion criteria were defined for study participation. The study was carried out from July 2017 to April 2018. The ethics committee of the Rhineland-Palatinate Chamber of Physicians approved study conduct.

### Survey

The survey items were developed according to a Europe-wide study on the delayed diagnosis of rare diseases [13]. For additional items (e. g., subjective health status), validated scales have been employed [14]. A two-stage pretest was conducted to ensure comprehensibility of the questionnaire. To prevent the small number of participants from being reduced, the pretest was performed on persons who were not affected by HAE.

### Study procedure

Study participants were recruited via three different approaches. First, patients were approached by attending physicians. Second, contact was established via patient advocacy groups. In addition, home therapy providers informed patients regarding the option to participate in this study. In case of interest, eligible patients were provided with further information on the study encompassing informative letters, a declaration of consent including information regarding the handling of data to be collected as well as appointment forms and the questionnaire. Patients willing to participate in the study contacted the IGES Institute with the signed declaration. The questionnaire was provided in advance in order to enable the participants to prepare for the interview (e. g., sorting documents, contacting family members). A trained interviewer carried out all telephone interviews. The survey items included questions regarding sociodemographic information, self-assessed health, clinical symptoms, utilization of healthcare services, and initial suspicion of the presence of a rare disease as well as the diagnostic process. Nearly all information collected related to the time before the diagnosis has been made. Each interview took an average of about 30 min.

### Statistical analysis

Descriptive analysis of the data obtained from the study population was performed. Proportional values as well as measures of central tendency and corresponding measures of dispersion were calculated.

## Results

Overall, 81 patients with HAE (74.1% female) were interviewed and included in the analysis. Table 1 shows characteristics of patients with HAE. Mean age of patients was 50.8 years (SD: 14.1). In most cases (78.8%), the person interviewed was the index patient (i. e., first patient in a family diagnosed with the disease). The majority of patients were either retired (44.4%) or still working (43.2%). Only a small proportion of those surveyed were incapacitated (3.7%).

**Table 1:** Characteristics of patients with hereditary angioedema (*n* = 81)

	Women (***n*** = 60)		Men (***n*** = 21)		All (n = 81)	
	n	%	n	%	n	%
**Age**						
0–19 years	1	1.7	0	0.0	1	1.2
20–39 years	11	18.3	1	4.8	12	14.8
40–59 years	26	43.3	5	23.8	31	38.3
60–79 years	18	30.0	12	57.1	30	37.0
≥ 80 years	4	6.7	3	14.3	7	8.6
All	60	100	21	100	81	100
Mean age (mean, SD)	53.8	15.2	65.2	13.4	50.8	14.1
**Index patient** ^a*^						
Yes	47	79.7	15	71.4	63	78.8
No	12	20.3	6	28.6	17	21.3
All	59	100.0	21	100.0	80	100.0
**Current occupation**						
Pupil / (university) student	4	6.7	0	0.0	4	4.9
Apprentice	0	0.0	0	0.0	0	0.0
Employed	28	46.7	7	33.3	35	43.2
Pensioner	22	36.7	14	66.7	36	44.4
Job-seeking	2	3.3	0	0.0	2	2.5
Incapacitated	3	5.0	0	0.0	3	3.7
Others	1	1.7	0	0.0	1	1.2
All	60	100.0	21	100.0	81	100.0

*SD* Standard deviation.

^**a**^ First person in the family to be diagnosed by hereditary angioedema

* *n* = 1 women with missing information

In the study, the patients were asked about their current state of health (“How would you describe your current state of health?”) and their state of health one year before diagnosis of HAE (“If you think of the year before the final diagnosis, how would you describe your state of health during that period?”).

As Table 2 shows, most of the patients reported their current health status as “good” (47.5%) or “very good” (13.8%). When comparing the current perceived state of health with that before the final diagnosis (“good” (16.3%) or “very good” (11.3%), it becomes clear that it has improved in most patients.

**Table 2:** Association between current state of health and the state of health one year before diagnosis in patients with hereditary angioedema (*n* = 81)

		State of health one year before diagnosis (self-rated)*													
		very good		good		fair		less well		poor		not specified		All	
		n	%	n	%	n	%	n	%	n	%	n	%	n	%
Current state of health (self-rated)	very good	2	*22.2*	3	*23.1*	4	*33.3*	1	*5.3*	1	*3.8*	0	0.0	11	13.8
	good	3	33.3	6	*46.2*	4	*33.3*	13	*68.4*	12	*46.2*	0	0.0	38	47.5
	fair	4	44.4	3	23.0	2	*16.7*	5	*26.3*	9	*34.6*	1	100	24	30.0
	less well	0	0.0	1	7.7	2	16.7	0	*0.0*	4	*15.4*	0	0.0	7	8.8
	poor	0	0.0	0	0.0	0	0.0	0	0.0	0	0.0	0	0.0	0	0.0
	not specified	0	0.0	0	0.0	0	0.0	0	0.0	0	0.0	0	0.0	0	0.0
	All	9	11.3	13	16.3	12	15.0	19	23.8	26	32.5	1	1.3	80	100

Note: Italicized  percentages indicate such constellations in which patients currently classify their state of health at least as well as in the year before the final diagnosis

*n = 1 patient with missing information

Furthermore, participants were asked to state which symptoms they suffered from before the correct diagnosis was made. In addition, the frequency of occurrence and the age of the first manifestation of the respective symptoms were assessed. Based on expert knowledge, a categorization of the described symptoms was carried out in order to ensure a uniform designation.

**Fig. 1:**
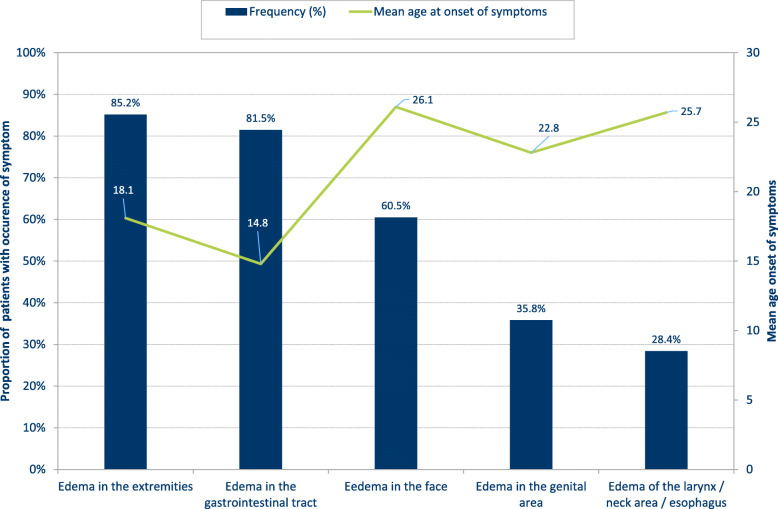
Prevalence and mean age at occurrence of the five most frequently reported symptoms in patients with hereditary angioedema (*n* = 81)

Participants most frequently reported angioedema in different localizations, which are typical for HAE: Angioedema in the extremities (85.2%), in the gastrointestinal tract (81.5%), in the face (60.5%), in the genital area (35.8%), and the laryngeal or neck area as well as in the esophagus (28.4%). These symptoms occurred first on average from early adolescence to early adulthood (14.8–26.1 years). The most common symptoms and the symptoms that manifested rather early in life, were edema in the extremities and edema in the gastrointestinal tract (Fig. 1). The mean age at first occurrence of any HAE-associated symptoms was 13.6 years, however, outliers led to a comparatively high mean age (median 10.0 years).

As stated earlier, unspecific symptoms and low awareness for rare diseases lead to delayed diagnoses. In the course of the diagnostic odyssey, patients can be misdiagnosed and potentially mistreated. Therefore, study participants were asked whether they were initially diagnosed differently due to the symptoms of HAE. Within this study, existence of misdiagnosis was assumed if diagnosis of another disease was made to explain HAE symptoms, at least for a short time. In contrast, a suspected diagnoses made during course of patient examination but not been confirmed, was not considered as a false diagnosis. The number of patients reporting misdiagnoses was *n* = 40 (49.4%). Table 3 shows most commonly misdiagnosed diseases.

**Table 3:** Misdiagnoses in patients with hereditary angioedema (*n* = 81)

	HAE (n = 81)	
	n	%
**Patients reporting misdiagnoses**		
Yes	40	49.4
No	41	50.6
All	81	100.0
**Most common misdiagnoses**^**a**^		
Appendicitis	16	40.0
Allergy	12	30.0
Mental disorder	6	15.0
Tonsilitis	3	7.5
Nervous stomach	3	7.5

^**a**^ Patients could have reported more than one misdiagnosis

*HAE* Hereditary angioedema

Of those having a false diagnoses, *n* = 32 (80%) reported a mistreatment. As shown in Table 4, initiated therapeutic measures were mainly pharmaceutical treatment (*n* = 21; 65.6%) or surgery (*n* = 18; 56.3%).

**Table 4:** Therapeutic measures initiated after misdiagnosis in patients with hereditary angioedema

	HAE (*n* = 40 with misdiagnoses)	
	n	%
**Number of patients reporting mistreatment**		
Yes	32	80.0
No	8	20.0
All	40	100.0
**Therapeutic measures**^**a**^		
Pharmaceuticals	21	65.6
Surgery	18	56.3
Others	3	9.4

*HAE* Hereditary angioedema

^a^ Patients could have reported alleged mistreatment without prior indication of a misdiagnosis

Subsequently, the patients provided information on the phase in which the presence of HAE was suspected for the first time and in which a confirmed diagnosis was made. The participants indicated that the suspicion of the disease was first reported on average at the age of 30.8 years (SD: 15.7 years). The first suspicion of HAE was most frequently expressed in a hospital (43.8%). In addition, a specialist was the second most frequent person group to raise the suspicion (18.8%). Final diagnosis was usually made shortly after the first suspicion had been raised (mean: 32.7 years, SD: 16.1).

Final diagnosis was most frequently made in a hospital (51.3%), by specialized centers (18.8%) and medical specialists (15.0%) or by a general practitioner (12.5%) (Table 5).

**Table 5:** Person group / institution making final diagnosis in patients with hereditary angioedema (*n* = 81)

	Women (n = 60)		Men (*n* = 20)		All (*n* = 80)	
	n	%	n	%	n	%
Person group / institution*						
General practitioner	9	15.0	1	5.0	10	12.5
Specialist	10	16.7	2	10.0	12	15.0
Specialized center	10	16.7	5	25.0	15	18.8
Hospital	29	48.3	12	60.0	41	51.3
Other health professions	0	0.0	0	0.0	0	0.0
Others	2	3.3	0	0.0	2	2.5
All	60	100.0	20	100.0	80	100.0

*SD* Standard deviation

*n = 1 male patient with missing information

From information on the first appearance of symptoms and the date of the final diagnosis, the diagnostic delay was calculated for each patient. The median duration of the diagnostic delay for HAE was 15.0 years (IQR: 23.0). Age-specific examination revealed a pronounced age-dependent gradient of the diagnostic delay, with persons in higher age groups also having a longer delay in diagnosis. In particular, people aged 60 had a significantly longer diagnostic delay. Furthermore, it could be observed that index patients had a notably longer diagnostic delay than family members who were diagnosed after the index patient. The median diagnostic delay of index patients was 19.0 years compared to 10.5 years in non-index patients (Table 6). The average time between HAE diagnosis and survey date was 18.1 years (women 24.0, SD: 14.5; men 21.2, SD: 21.0). There was no significant difference in the patients’ assessment of the state of health and symptoms before diagnosis in relation to the time elapsed since diagnosis. This may seem surprising at first glance, but in our opinion it can be explained by the incisive diagnosis, the concomitant circumstances of which most patients have vivid memories for a long time according to their statements in the survey.

**Table 6:** Diagnostic delay of patients with hereditary angioedema (*n* = 81)

	HAE (*n* = 81)				
	n	Mean	SD	Median	IQR
**Diagnostic delay (in years)**					
Overall*	78	18.1	14.6	15.0	23.0
By age group*					
0–19 years	1	2.0	/	2.0	/
20–39 years	12	8.0	5.3	7.0	7.8
40–59 years	30	15.0	12.3	12.0	19.5
60–79 years	29	22.9	13.8	24.0	19.5
≥ 80 years	6	38.5	18.6	44.0	33.8
All	78				
By index patient**					
Yes	61	19.4	15.0	19.0	24.0
No	16	13.1	12.0	10.5	14.3
All	77				

“/”: No values available due to low case numbers.

*SD* Standard deviation, *IQR* Interquartile range, *HAE* Hereditary angioedema

*n = 3 patients with missing information

**n = 4 patients with missing information

## Discussion

In the present study, 81 patients with HAE were asked about the phase from the onset of the first symptoms to the final diagnosis in order to trace their “Patient Journey” through the German healthcare system. With regard to the sociodemographic characteristics of the participants (age, gender, level of education), a partly differential picture emerges against the background of previous research on HAE. The calculated mean age of 50.8 (SD 14.1) years in our study is comparable to other studies to a limited extent, since publications based on disease or treatment registers usually report the age for inclusion in the register. Since inclusion usually occurs either at the time of diagnosis or treatment initiation, the reported age is correspondingly lower than in the present study. For example, the corresponding mean age in the Icatibant Outcome Survey (IOS) was 46.6 years [12]. The higher mean age in the present study could be due to a lower willingness of younger people to participate and because older patients are more often involved in patient advocacy groups which was the main source of recruitment in this study (80% of HAE patients). In relation to gender distribution, the proportion of women was comparatively high (74.1%). In the IOS, the corresponding share was 62.1% [12]. The higher proportion of female HAE patients in the presented study may be attributable to a higher interest of women in the specific study topic. For instance, women show a more severe course of the disease that is characterized in particular by more frequent swelling [15].

One of the main focusses of this study was the description of the diagnostic delay in HAE patients. In our study, the median diagnostic delay was 15 years (IQR: 23.0). Compared to other studies, this diagnostic delay is relatively long. According to the IOS, patients with HAE showed an average diagnostic delay of 8.5 years [7]. The long diagnostic delay can be attributed to the high proportion of index patients within the present study as well as to the higher age of the patients. Whereas the proportion of index patients in the present study was 61%, the results of the IOS, for example, showed a proportion of 14.7% index patients [16]. Index patients are of particular interest for research questions regarding the diagnostic delay, because these persons have to go through the diagnostic process without prior knowledge within the family. Thus, a longer period usually elapses before a reliable diagnosis is made. In line with this, a subgroup analysis showed that index patients had a longer diagnostic delay (Median: 19.0, IQR: 24.3) as compared to patients who were not the first to be diagnosed with HAE in their extended family (Median: 10.5, IQR: 14.3).

However, also non-index patients had a remarkable diagnostic delay. This may be astonishing at first sight. From clinical expertise it not self-evident that in a family all members are informed about diseases diagnosed in relatives. Due to a lack of communication in the family, it is quite possible that the occurrence of symptoms is not promptly associated with those of the index patient in the family. This would only happen if the family knew that the disease was hereditary, which is not always the case. In addition, because of a lack of awareness of HAE, doctors who are consulted when symptoms occur may not immediately conclude that these symptoms may be due to hereditary disease. It can be assumed that patients who have experienced a longer diagnostic delay were probably more interested to participate in the study. Interpretation of results on the diagnostic delay should therefore take into account a possible selection of specific patients within the present study.

Subgroup analyses also revealed an age-dependent gradient of diagnostic delay. Older patients showed a longer diagnostic delay than younger participants did. For example, the median diagnostic delay in the 60 to 79 age group was 24 years, while the 20 to 39 age group had a median diagnostic delay of 7 years. The longer diagnostic delay in older age groups is probably largely explained by low awareness of orphan diseases in former decades. The lower awareness in turn can be explained by the unavailability of effective pharmaceuticals for the treatment of HAE during these former decades. In the IOS, for example, it has been shown that the proportion of patients with HAE receiving false diagnoses, which are closely linked to diagnostic delay, decreased significantly in the course of the patients’ birth decades [16].

Once the final diagnosis is made and uncertainty about the existing symptoms has been resolved, patients are likely to reassess their life situation. A further focus of our study was therefore on the current quality of life of the HAE patients surveyed. With regard to the self-assessed state of health, the participants drew a predominantly positive picture. The majority of patients (61.3%) rated their current state of health as “very good” or “good”, compared to only 27.6% before the diagnosis HAE was made. When putting the results into the research context, it is important to consider the characteristic values of the scale employed. In the present study, “fair” was offered as a category alongside “very good”, “good”, “less well” and “poor”. A representative survey of the general German population conducted by the University of Leipzig, which also used “fair” as the middle category, shows that 60.8% of respondents evaluated their state of health as “good” or “very good” [17]. Based on these results, it can be assumed that patients diagnosed with HAE assess their state of health similar to that of the reference population. This initially surprising result may be related to the fact that effective therapeutic options for HAE, allowing adequate prophylactic and acute symptom control, have now been developed [18].

In order to understand how the health-related well-being of HAE patients is perceived, it is necessary to consider the symptoms. The types of symptoms reported prior to diagnosis (e. g., angioedema with different localizations) and their prevalence are well consistent with observations from other studies [15]. Since the symptoms present as acute episodes that are painful and often visible, underestimation of these symptoms is unlikely. Nevertheless, these symptoms are not disease-specific so that physicians without extensive knowledge of this rare disease could directly attribute them to HAE. Thus, misdiagnosis is common. Reliable information on the frequency of misdiagnosis due to symptoms of HAE is already available within the framework of the IOS. Here, about 44% of patients with HAE have been misdiagnosed before getting HAE diagnosis [16]. In the present study, the proportion of patients with false diagnoses was approx. 50% and thus somewhat higher. One explanation for this lies in the disproportionately large number of index patients who participated in the present study. As already mentioned before, index patients exhibit longer diagnostic delays and have thus a higher probability of misdiagnosis. However, the type of false diagnoses corresponds to the results of the IOS. As in the present study, appendicitis and allergies were the most frequently reported misdiagnosed conditions [16]. It should be mentioned that a conservative definition of false diagnoses was chosen in the present study. Suspected diagnoses that could not be substantiated in the further course of the medical investigation were not regarded as false diagnoses. However, cases in which it was assumed that the symptoms were due to another disease were regarded as misdiagnosis.

In the present study, the first suspicion of the diagnosis HAE was most frequently expressed in a hospital (43.8%). The final diagnosis was also most often made in a hospital (51.3%), followed by specialized centers (18.8%). It should be mentioned that patients in our study probably did not discriminate between specialized centers and hospitals, but wrongly classified e. g. a “normal” dermatological department of a hospital as a specialized HAE centre. Therefore, the proportion of final diagnoses made in a specialized centre is likely to be overestimated, while the proportion of final diagnoses in hospitals is likely to be underestimated. However, since these are distinct categories, the single proportional values can be added together.

### Strengths and limitations

In general, the percentage of index patients covered in this survey is relatively high. This may indicate a noticeable bias in the study population. Apparently, index patients are the ones who were more willing to participate in the study. This may be due to the fact that the study approach – namely to show the way to diagnosis – has particularly appealed to these patients, as they themselves have had to go through a long odyssey before the correct diagnosis was made. On the other hand, when a child of an already diagnosed HAE patient is diagnosed, the period of uncertainty is usually shorter and is not marked by spectacular aberrations by the health care system. In this respect, selective participation of index patients, older patients and those who had a relatively long diagnostic delay in general can be assumed leading to a somewhat restricted external validity of the results. Rather, since an overrepresentation of patients who have an increased interest in the study subject because of their own “Patient Journey” can be assumed some results differ from findings of other studies which is particularly true for the duration of the diagnostic delay and the frequency of misdiagnoses. When interpreting the results, it must also be kept in mind that the information was collected as part of a survey. It is therefore possible that due to a recall bias, information or recollections of past events have not been reported or reproduced adequately. In particular, the reported symptom burden prior to final diagnosis and the utilization of the health care system prior to correct diagnosis are likely to be underestimated.

## Conclusion

The diagnostic delay is an important issue for HAE patients and particularly for index patients, since especially misdiagnosis and subsequent treatment due to misdiagnosis has major implications for patients. This study showed that self-perceived status of health for patients is much better once the final correct diagnosis has been made and specific treatment was available. The results show that final diagnosis is mainly made in hospital or specialist centers. Further challenge in the future will still be to increase awareness for these diseases especially in settings which are normally approached by patients at occurrence of first symptoms to assure early referral to specialists and therefore increase the likelihood of receiving an early diagnosis.

## Data Availability

The datasets generated and analyzed during the current study are not publicly available due the fact that the informed consent only included publication of data on an aggregated level and not on an individual (anonymized) level. Respective precautions had to be taken to minimize the risk of re-identification of individual patients.
