# Correction to: A Germany-wide survey study on the patient journey of patients with hereditary angioedema

**DOI:** 10.1186/s13023-021-01714-7

**Published:** 2021-02-22

**Authors:** Markus Magerl, Holger Gothe, Simon Krupka, Anja Lachmann, Christoph Ohlmeier

**Affiliations:** 1 Dermatological Allergology, Allergie-Centrum-Charité, Department of Dermatology and Allergy, Charité – Universitätsmedizin Berlin, Luisenstraße 2, 10117 Berlin, Germany; 2 IGES Institut GmbH, Friedrichstraße 180, 10117 Berlin, Germany; 3 Chair for Health Sciences/Public Health, Medical Faculty “Carl Gustav Carus”, Technical University Dresden, Loescherstrasse 18, 01307 Dresden, Germany; 4 Institute of Public Health, Medical Decision Making and Health Technology Assessment, Department of Public Health, Health Services Research and Health Technology Assessment, UMIT – University for Health Sciences, Medical Informatics and Technology, Eduard Wallnoefer Zentrum 1, 6060 Hall in Tirol, Austria; 5 Shire Deutschland GmbH, Friedrichstraße 149, 10117 Berlin, Germany

## Correction to: Orphanet Journal of Rare Diseases (2020) 15:221 https://doi.org/10.1186/s13023-020-01506-5

In the original version of this article [1], the affiliations of authors Holger Gothe and Anja Lachmann were unfortunately interchanged:

Dr. Gothe was erroneously presented as affiliated with the Shire Deutschland GmbH, while Dr. Lachmann as affiliated with the Chair for Health Sciences/Public Health, Medical Faculty "Carl Gustav Carus", Technical University Dresden.

The correct authors’ affiliations are shown here below and are included in the ‘Author details’ section of this Correction. These have already been corrected in the original article.

Holger Gothe^2,3,4^

2 IGES Institut GmbH, Friedrichstraße 180, 10117 Berlin, Germany.

3 Chair for Health Sciences / Public Health, Medical Faculty "Carl Gustav Carus", Technical University Dresden, Loescherstrasse 18, 01307 Dresden, Germany.

4 Institute of Public Health, Medical Decision Making and Health Technology Assessment, Department of Public Health, Health Services Research and Health Technology Assessment, UMIT – University for Health Sciences, Medical Informatics and Technology, Eduard Wallnoefer Zentrum 1, A-6060 Hall in Tirol, Austria.

Anja Lachmann ^5^.

5 Shire Deutschland GmbH, Friedrichstraße 149, 10117 Berlin, Germany.

## References

[1] Magerl et al. A Germany-wide survey study on the patient journey of patients with hereditary angioedema. Orphanet J Rare Diseases. 2020;15:221. 10.1186/s13023-020-01506-5. 10.1186/s13023-020-01506-5 PMC7448463 32843072

